# Digital PCR (dPCR) Quantification of miR-155-5p as a Potential Candidate for a Tissue Biomarker of Inflammation in Rabbits Infected with *Lagovirus europaeus*/Rabbit Hemorrhagic Disease Virus (RHDV)

**DOI:** 10.3390/v15071578

**Published:** 2023-07-19

**Authors:** Beata Hukowska-Szematowicz, Ewa Ostrycharz, Wioleta Dudzińska, Paulina Roszkowska, Aldona Siennicka, Iwona Wojciechowska-Koszko

**Affiliations:** 1 Institute of Biology, University of Szczecin, 71-412 Szczecin, Poland; ewa.ostrycharz@phd.usz.edu.pl; 2 Molecular Biology and Biotechnology Center, University of Szczecin, 71-412 Szczecin, Poland; 3 Doctoral School, University of Szczecin, 71-412 Szczecin, Poland; 4 Department of Functional Diagnostics and Physical Medicine, Pomeranian Medical University in Szczecin, Żołnierska 54, 71-210 Szczecin, Poland; wioleta.dudzinska@pum.edu.pl; 5 Department of Diagnostic Immunology, Pomeranian Medical University, Powstańców Wielkopolskich 72, 70-111 Szczecin, Poland; paulina.roszkowska@pum.edu.pl (P.R.); iwona.koszko@pum.edu.pl (I.W.-K.); 6 Department of Laboratory Diagnostics, Pomeranian Medical University, Powstańców Wielkopolskich 72, 70-111 Szczecin, Poland; aldona.siennicka@pum.edu.pl

**Keywords:** digital PCR (dPCR), microRNA-155 (miR-155), biomarker, inflammation, Rabbit Hemorrhagic Disease Virus (RHDV), tissues

## Abstract

MicroRNAs (miRNAs, miRs) are a group of small, 17–25 nucleotide, non-coding RNA sequences that, in their mature form, regulate gene expression at the post-transcriptional level. They participate in many physiological and pathological processes in both humans and animals. One such process is viral infection, in which miR-155 participates in innate and adaptive immune responses to a broad range of inflammatory mediators. Recently, the study of microRNA has become an interesting field of research as a potential candidate for biomarkers for various processes and disease. To use miRNAs as potential biomarkers of inflammation in viral diseases of animals and humans, it is necessary to improve their detection and quantification. In a previous study, using reverse transcription real-time quantitative PCR (RT-qPCR), we showed that the expression of ocu-miR-155-5p in liver tissue was significantly higher in rabbits infected with *Lagovirus europaeus*/Rabbit Hemorrhagic Disease Virus (RHDV) compared to healthy rabbits. The results indicated a role for ocu-miR-155-5p in *Lagovirus europaeus*/RHDV infection and reflected hepatitis and the impairment/dysfunction of this organ during RHD. MiR-155-5p was, therefore, hypothesized as a potential candidate for a tissue biomarker of inflammation and examined in tissues in *Lagovirus europaeus*/RHDV infection by dPCR. The objective of the study is the absolute quantification of ocu-miR-155-5p in four tissues (liver, lung, kidney, and spleen) of rabbits infected with *Lagovirus europaeus*/RHDV by digital PCR, a robust technique for the precise and direct quantification of small amounts of nucleic acids, including miRNAs, without standard curves and external references. The average copy number/µL (copies/µL) of ocu-miRNA-155-5p in rabbits infected with *Lagovirus europaeus* GI.1a/Rossi in the liver tissue was 12.26 ± 0.14, that in the lung tissue was 48.90 ± 9.23, that in the kidney tissue was 16.92 ± 2.89, and that in the spleen was 25.10 ± 0.90. In contrast, in the tissues of healthy control rabbits, the average number of copies/µL of ocu-miRNA-155-5p was 5.07 ± 1.10 for the liver, 23.52 ± 2.77 for lungs, 8.10 ± 0.86 for kidneys, and 42.12 ± 3.68 for the spleen. The increased expression of ocu-miRNA-155-5p in infected rabbits was demonstrated in the liver (a fold-change of 2.4, *p*-value = 0.0003), lung (a fold-change of 2.1, *p*-value = 0.03), and kidneys (a fold-change of 2.1, *p*-value = 0.01), with a decrease in the spleen (a fold-change of 0.6, *p*-value = 0.002). In the study of *Lagovirus europaeus*/RHDV infection and in the context of viral infections, this is the first report that shows the potential use of dPCR for the sensitive and absolute quantification of microRNA-155-5p in tissues during viral infection. We think miR-155-5p may be a potential candidate for a tissue biomarker of inflammation with *Lagovirus europaeus*/RHDV infection. Our report presents a new path in discovering potential candidates for the tissue biomarkers of inflammation.

## 1. Introduction

MicroRNAs (miRNAs or miRs) are small non-coding RNAs (17–25 nucleotides) that act as post-transcriptional regulators of gene expression [1]. It has been shown that miRNAs participate in many physiological and pathological processes in both humans and animals [2,3]. One of these processes is viral infection [4]. In this context, there is a group of key host miRNAs involved in viral infections in humans and animals [4,5]. One such molecule is miR-155, which participates in innate and adaptive immune responses to a wide range of inflammatory mediators [4,6,7,8,9]. In the course of viral infections, miRNA-155 regulates innate immunity by influencing the interferon (IFN) response, natural killer (NK) cell activity, or macrophage polarization, and also affects adaptive immunity, e.g., regulates the production of antiviral antibodies and T lymphocytes, including cytotoxic T lymphocytes (CTLs), T helper (Th1, Th2, Th17) cells, follicular helper T (Tfh) cells, and regulatory T cells (Tregs) [5,10].

*Lagovirus europaeus* (*L. europaeus*)/Rabbit Hemorrhagic Disease Virus (RHDV) [11] is the etiological agent of rabbit hemorrhagic disease (RHD) [12,13]. Within one to three days after infection via oral or nasal route transmission in animals, rabbits manifest the first clinical signs, such as apathy, fever, and respiratory disorders [12,13]. The highest titer of *L. europaeus*/RHDV is found in the liver, spleen (reported in the presence of the chronic or sub-acute forms of the disease), lungs, kidneys, and bone marrow, which are the most frequently assessed organs in the diagnostic process [12,14,15]. *L. europaeus*/RHDV can also be detected in biological fluids such as serum, urine, and feces samples [15]. During the development of RHD, many pathological changes occur in rabbit organs, especially in the liver (the site of viral replication), lungs, kidneys, spleen, and trachea [12]. The main changes in RHD are the acute inflammation of the liver, spleen, lungs, and kidneys, with hemorrhage and congestion in the lungs, heart, and kidneys. Inflammatory foci rich in neutrophils and T and B lymphocytes are found in the liver, lungs, spleen, and kidneys, and the activation of Kupffer cells occurs in the liver and alveolar macrophages in the lungs [14,16,17,18]. Disease progression is correlated with increased apoptosis of hepatocytes and liver endothelial cells [19,20], as well as necrosis [21]. The pathogenesis of the disease also includes the apoptosis of T and B cells in the liver, spleen and peripheral blood [22,23,24,25,26], and a decrease in regulatory T cells [27]. Innate and adaptive immunity also play an important role in the pathogenesis of RHD [22,24,28,29,30,31,32], including of peripheral blood leukocytes [33,34,35]. After infection with *L. europaeus*, an increase in pro-inflammatory and anti-inflammatory cytokines occurs in the serum, liver, and spleen [27,33,34,36,37,38].

In a previous publication, we showed that the signature of miRNAs in viral infections in humans and animals is similar and includes several miRNAs. The best-described sequence is miR-155 [4,39]. Studies have indicated that miR-155 plays a key role in inflammation that can be observed across many different human and animal tissue systems [7]. The association of miR-155 with both *L. europaeus* infection and RHD is based on the increased expression of this miR in the liver (other organs have not been studied to date) and inflammatory cells infiltrating this organ, which may be crucial for an adequate inflammatory response in the damaged liver. In addition, a functional analysis showed that ocu-miR-155-5p could regulate the expression of genes involved in processes correlated with acute liver failure (ALF) in rabbits [39].

MiRNAs were first established as biomarkers for cancer in 2008, when Lawrie et al. [40] utilized them for the examination of diffuse, large B cell lymphoma in the serum of patients. Ever since, their potential use as biomarkers has been mentioned in the literature for numerous diseases [41], including viral diseases [42,43,44,45,46,47,48,49,50,51]. In this paper, we indicate that *L. europaeus* infection in rabbits is a good research model and a signpost for the research of miRNAs as potential candidates for a tissue biomarker of inflammation in viral infection.

There are currently no good non-protein biomarkers to predict specific immune events, such as inflammation. MiRNA-type tissue biomarkers in animals and humans could function as stand-alone specific markers due to the fact that miRNA tissue profiles can help to study local immune responses better than peripheral ones. They could also serve as biomarkers to confirm a diagnosis after the examination of other peripheral biomarkers of inflammation and additional circulating miRNAs. In the case of the latter, they are known to be stable, present in various biological fluids during various diseases, and may act as biomarkers [41,52]. In contrast, very little is known about miRNAs from tissues and their potential use as tissue biomarkers of disease. Using in silico analysis, Ciu and Ciu [52] showed that miRNA communication between tissues and body fluids often occurs, and potential miRNA signatures in tissues and body fluids are not random but follow regular patterns. In the case of miRNAs from tissues, it is debatable whether they meet the biomarker criterion. Biomarkers should be sensitive, robust, easy to detect, and provide information about the examined process. Lastly, they should be translatable from research to the clinic [41]. In our opinion, they meet most of the criteria, whereas the practical aspects and advantages of tissue harvesting (biopsy) for determining tissue miRNA biomarkers in both diseased rabbits and humans are supported by the following arguments: (1) MiRNA tissue profiles may help to study local immune responses better than peripheral ones. In the case of circulating miRNAs, it is uncertain where they come from. MiRNAs from tissues can help in the earlier diagnosis of specific organs and ongoing pathological processes. (2) MiRNA tissue profiles could help in distinguishing between acute and sub-acute phases of the disease, prognosis, and possibly even predicting mortality. (3) MiRNAs from tissues show high specificity in biological processes and are sensitive, as they vary with the process/disease progression. (4) Technologies for detecting miRNAs from tissues already exist. New methods, such as dPCR, require less time and lower costs to develop compared to producing new antibodies for protein biomarkers.

To use miRNAs as potential biomarkers of inflammation in viral diseases of animals and humans, it is necessary to improve their detection and quantification. In a previous study, using quantitative real-time reverse transcription PCR (RT-qPCR), we showed that the expression of ocu-miR-155-5p in the liver tissue was significantly higher in RHDV-infected rabbits compared to healthy rabbits. The results indicated a role for ocu-miR-155-5p in RHDV infection and reflected hepatitis and the impairment/dysfunction of this organ during RHD [39]. MiR-155-5p was, therefore, hypothesized as a potential candidate for a tissue biomarker of inflammation and examined in various tissues in *Lagovirus europaeus*/RHDV infection by dPCR.

**Figure 1:**
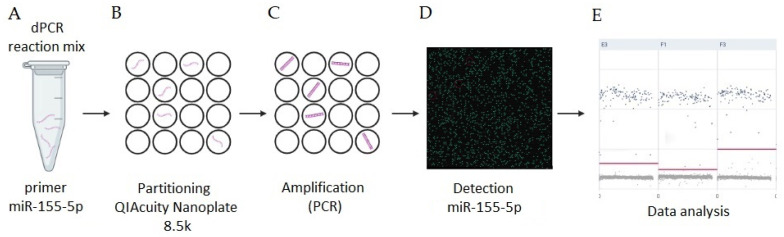
Scheme of the digital PCR (dPCR) quantification of microRNA (miR-155-5p) in the tissues of rabbits infected with *Lagovirus europaeus* (*L. europaeus*)/Rabbit Hemorrhagic Disease Virus (RHDV). The research used the QIAcuity Digital PCR System, Qiagen, Hilden, Germany. (**A**) Sample preparation (dPCR reaction mix containing the target sequences miR-155-5p and background; see the Methods Section). (**B**) Partitioning (performed on a special 8.5k nanoplate; the sample is divided into many independent partitions such that each contains one or no target sequences of interest). (**C**) Amplification (PCR) (each partition acts as an individual PCR microreactor, amplifying the target sequences in each of them). (**D**) Detection (partitions that contain the amplified target miR-155-5p are detected by fluorescence). The ratio of positive partitions (presence of fluorescence) over the total number allows for the determination of the concentration of the target in the sample. (**E**) Data analysis (data output from dPCR assay to the absolute quantification of miR-155-5p in rabbit tissues infected with *L. europaeus*). One-dimensional scatterplots of event number–droplets vs. fluorescence amplitude for an ideal assay with a clear separation of positive (blue) and negative (grey) droplets.

Digital PCR (dPCR) is a technique that allows for the absolute quantification of the low-abundance nucleic acids, including microRNAs [53,54,55,56,57,58,59,60], without the need for an external reference or standard curve, and is more reproductible than real-time PCR since it is less affected by variations in PCR efficiency [61,62,63]. The principle of dPCR is based on partitioning, i.e., the separation of the dPCR reaction mixture into thousands of independent partitions, thanks to the use of special microwell plates (Figure 1) [64,65]. By separating the reaction mixture into thousands of partitions, it is possible to study each single molecule [61]. Due to the aforementioned division of the mixture, the concentration of the target molecules is reduced by several orders of magnitude, which affects the correct amplification of the desired targets, i.e., there is a higher sensitivity of the reaction. In addition, divisions of the reaction mixture reduce the number of inhibitors in each unit, which results in a better reaction stability [61,63,64].

The dPCR method has many advantages, such as absolute quantitative assessment [66], and, compared with real-time PCR, it is characterized by a higher sensitivity, stability, and precision, enabling the detection of amounts of the tested molecules that are on the verge of the detection sensitivity, using the previously mentioned methods, and even allows the detection of a single molecule, including one miR sequence [64,66].

The aim of this study is the absolute quantification of ocu-miR-155-5p in four tissues, liver, lung, kidney, and spleen, of rabbits infected with *L. europaeus*/RHDV by dPCR. In the study of *L. europaeus* infection, and in the context of viral infections, this is the first report that explores the potential use of dPCR for the precise and sensitive quantification of host miRNAs in tissues during a viral infection.

## 2. Materials and Methods

### 2.1. Materials

#### 2.1.1. Animals

The study was performed on 20 healthy, conventional mixed-breed rabbits of both sexes of *Oryctolagus cuniculus* [67].

The animals were purchased from licensed breeders, and they were kept under constant veterinary and zoo hygienic supervision during the experiment [68]. During the experiment, appropriate zoo technical conditions were ensured, in accordance with the recommended Polish standards developed in line with the European Union Directive with regard to temperature and humidity, as well as the lighting and size of cages for animals [69]. The animals were not vaccinated against *L. europaeus*; nevertheless, before the experiment, all animals were tested for anti-RHD antibodies using an ELISA test (Instituto Zooprofilattico Sperimentale, Teramo, Italy). The experiment was approved by the Local Ethics Committee in Szczecin (no. 1/2009, 26.01.2009). During the experiment, the clinical conditions of the infected and healthy control rabbits were assessed.

#### 2.1.2. *Lagovirus europaeus*/RHDV

In the experiment, a variant of *L. europaeus*/RHDV was used, GI.1a/Rossi (Germany, 2000). The rabbits were divided into two groups: (I) healthy control rabbits (n = 10) and (II) rabbits infected with *L. europaeus* GI.1a/Rossi (n = 10). The experimental animals were infected by an intramuscular injection of the virus of 1 mL, while the healthy control rabbits were injected with PBS (phosphate-buffered saline) as a placebo. The virus was obtained from liver samples from rabbits that had died naturally. A liver homogenate was prepared from the recovered livers, and the rabbits were experimentally infected to increase the amount of virus. After their death, the liver was prepared as a 20% homogenate, purified by centrifugation (3000 rpm), treated with 10% chloroform for 60 min, and centrifuged again. Then, a 1:1 glycerol suspension was prepared [32,70]. The prepared antigen had viral particles with a density of 1310 g/cm^3^.

#### 2.1.3. Tissue Samples

Tissue samples from the liver, lungs, spleen, and kidneys were obtained from the infected animals (n = 10 rabbits) post-mortem, clinically defined. The organs were taken from the healthy control animals (n = 10 rabbits) after euthanasia. Each tissue sample was washed in cold PBS and immediately placed in liquid nitrogen. Tissue samples were stored at −80 °C until miRNA extraction.

### 2.2. Methods

#### 2.2.1. MiRNA Isolation from the Tissue Samples and Reverse Transcription Reaction

MiRNA was extracted from 50 mg of each tissue sample of the infected and healthy rabbits using an miRNeasy Mini Kit (Qiagen, Hilden, Germany) according to the manufacturer’s protocol. The quality and quantity of the isolates were assessed using a NanoDrop 2000 spectrophotometer (Thermo Fisher Scientific, Walthman, MA, USA).

cDNA synthesis from the obtained miRNA was conducted with an miRCURY LNA RT Kit (Qiagen, Hilden, Germany). The input amount of template RNA for reverse transcription was 5 ng/µL and the concentration was measured using a NanoDrop 2000 spectrophotometer (Thermo Fisher Scientific, Waltham, MA, USA).

The reaction mixture consisted of 2 μL 5× miRCURY SYBR Green RT Reaction Buffer, 1 μL 10× miRCURY RT Enzyme Mix, 4.5 μL RNase-free water, and 2 μL of template RNA with the addition of UniSp6 RNA spike-in (Qiagen, Hilden, Germany), according to the manufacturer’s recommendation. The reaction was conducted in the following steps: (i) reverse transcription step by incubation at 42 °C for 60 min, (ii) inactivation of the reaction at 95 °C for 5 min, and (iii) immediate cooling to 4 °C on Mastercycler PRO S (Eppendorf, Hamburg, Germany). The cDNAs obtained were stored at −20 °C for further analysis.

#### 2.2.2. Quantification of Ocu-miR-155-5p in Tissue Samples Using dPCR

The expression of ocu-miR-155-5p in tissue samples was measured by the QIAcuity One dPCR System (Qiagen, Hilden, Germany). The reactions were conducted using miRCURY LNA PCR Assays (10×), ocu-miR-155-5p (Qiagen, Hilden, Germany), 3× EvaGreen PCR Master Mix (Qiagen, Hilden, Germany), template cDNA, and RNase-free water. For the dPCR reaction, the cDNA was diluted 1:60 for miRNA detection. The reaction mixture together with the cDNA template in a total volume of 12 µL was applied to the QIAcuity Nanoplate 8.5k (24-well) (Qiagen, Hilden, Germany). The plate was sealed using the foil provided in the QIAcuity Nanoplate Kit (Qiagen, Hilden, Germany). The thermal cycling conditions were as follows: (I) PCR initial heat activation 95 °C for 2 min, (II) 40 cycles of denaturation 95 °C for 15 s and annealing 60 °C for 1 min, and a (III) final step of cooling down 40 °C for 5 min. To check the efficiency of the dPCR reaction, a control plate assay was performed using a UniSP6 (Qiagen, Hilden, Germany); to check the purity of the performed reaction, a no-template control (NTC) was conducted. For the analysis of the fluorescence data, QIAcuity Software Suite (Qiagen, Hilden, Germany) with green channel was used. The images were exposed for 400 ms with a gain of 4. The final concentration of miRNA is expressed in unit copies/μL.

#### 2.2.3. Statistics

The obtained results were subjected to statistical analysis. A Shapiro–Wilk test was performed to evaluate the agreement of the samples with the normal distribution. This analysis showed that the data for the liver, lung, and spleen did not deviate from the normal distribution, while the data for the kidney deviated from the normal distribution. In order to check whether there was a significant change in the level of ocu-miR-155-5p in the organs of the infected rabbits compared to healthy rabbits, Student’s *t*-test was performed for independent samples of the liver, lung, and spleen. For the kidney, a Mann–Whitney U-test was performed for independent samples. The results were considered statistically significant with a *p*-value equal to or lower than 5%. The examined parameters were additionally characterized by the standard error. The results were analyzed using the statistical package Statistica PL 13 (StatSoft Kraków, Kraków, Poland).

## 3. Results

### 3.1. Absolute Quantification of Ocu-miRNA-155-5p by dPCR in Rabbits Infected with L. europaeus/RHDV

DPCR was used to quantify the copy number of ocu-miR-155-5p in four tissues—liver, lung, kidney, and spleen of *L. europaeus*-infected rabbits and in the same tissues of healthy control rabbits.

**Figure 2:**
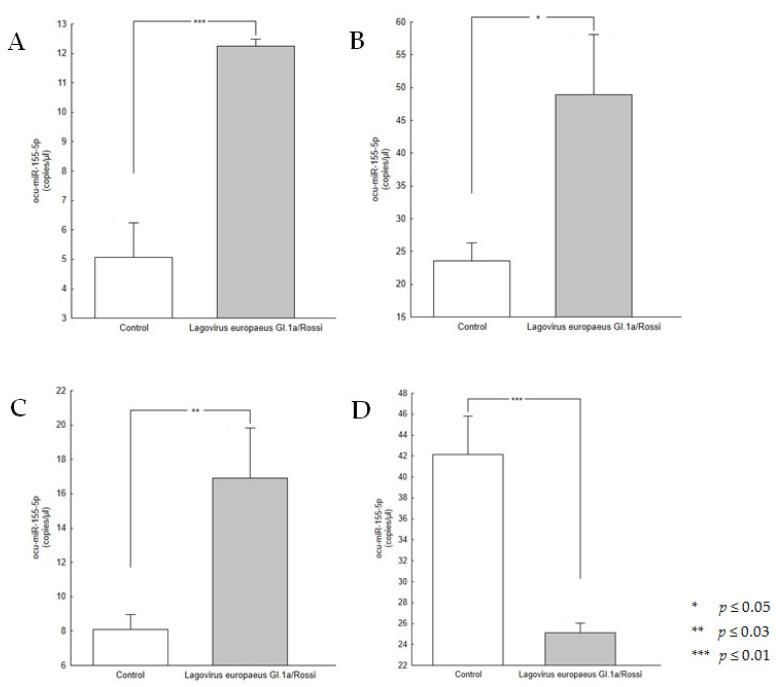
Absolute quantification of ocu-miR-155-5p by dPCR. Liver (**A**), lung (**B**), kidney (**C**), and spleen (**D**). Data are shown as the mean ± standard error (SE), and *p* ≤ 0.05 was considered significant.

The studies showed that the average copy number/µL (copies/µL) of ocu-miR-155-5p in rabbits infected with *L. europaeus*, variant GI.1a/Rossi, in the liver tissue was 12.26 ± 0.14 (Figure 2A), in the lung tissue was 48.90 ± 9.23, (Figure 2B), in the kidney was 16.92 ± 2.89 (Figure 2C), and in the spleen was 25.10 ± 0.90 (Figure 2D). In contrast, in the tissues of the healthy control rabbits, the average copy number/µL (copies/µL) of ocu-miR-155-5p was 5.07 ± 1.10 for the liver, 23.52 ± 2.77 for the lung, 8.10 ± 0.86 for the kidney, and 42.12 ± 3.68 for the spleen (Figure 2A–D).

Comparing the results obtained in the group of infected rabbits to the results in the group of control rabbits, an increase in the expression of ocu-miR-155-5p was found in infected rabbits in the liver (a fold-change of 2.4, *p*-value = 0.0003), lungs (a fold-change of 2.1, *p*-value = 0.03), and kidneys (a fold-change of 2.1, *p*-value = 0.01), while there was a decrease in the spleen (a fold-change of 0.6, *p*-value = 0.002).

### 3.2. Clinical Signs of Lagovirus europaeus/RHDV Infection

During the experiment, the animals showed clinical signs of RHD, including apathy, gelled feces, no response to external stimuli, and rapid breathing. The mortality rate after infection by day 4 of the experiment was 40%.

## 4. Discussion

MiRNA-155 is one of the best-described miRNA sequences [71]. It participates in maintaining the homeostasis of the cells of the immune system, and in the immune response and inflammation in the course of various disease entities [71]. MiR-155 expression was first described in the human liver, lung, kidney, spleen, and thymus [8]. In the context of immune response, miR-155 is expressed in various cell types, including B lymphocytes and T lymphocytes and their subpopulations, granulocytes, monocytes, macrophages, NK cells, and dendritic cells (DCs). In these cells, it regulates the secretion of cytokines and chemokines [6,8]. MiR-155 is rapidly upregulated by nuclear factor-κB (NF-κB) within the first 12 h of inflammatory response [7,8]. MiR-155 is particularly responsive to many inflammatory stimuli, such as tumor necrosis factor alpha (TNF-α), interleukin (IL)-IL-1β, interferons (IFNs), pathogen-associated molecular patterns (PAMPs) and damage-associated molecular patterns (DAMPs), alarmins (e.g., IL-1α), and hypoxia, as well as to Toll-like receptor (TLR) ligands in various cell types, particularly in monocytes/macrophages [8]. MiR-155-5p in humans and animals is the dominant form, and is particularly important for the regulation of the inflammatory response [8,72].

In a previous study that used RT-qPCR, we showed that the expression of ocu-miR-155-5p in the liver tissue was significantly higher in RHDV-infected rabbits compared to in healthy rabbits [39]. In view of the above fact, we hypothesized that miR-155-5p could be a potential candidate for a tissue biomarker of inflammation, and we examined this sequence in four tissues in *L. europaeus*/RHDV infection by dPCR.

In our study, a digital PCR was proposed as a powerful method for the precise and absolute quantification of miRs in tissues and offers significant advantages over standard real-time PCR, i.e., higher sensitivity and reproducibility, mainly because it does not require standard curves and normalization [62,64,66]. The disadvantage of the method is the high purchasing cost of the system (QIAcuity Digital PCR System, Qiagen, Germany), as well as of the plates, assays, and kits required for determination. Previous research has used the dPCR method in molecular diagnostics [73], personalized medicine [62], and virology [74,75] and has mainly been limited to the quantitative determination of viruses [75,76,77,78,79,80,81,82]. For some time, this method has also been used in miR studies related to the quantification of this molecule in various types of cancer [53,54], bacterial diseases [55], and neurological [56], cardiovascular [57], and neuromuscular disorders [58]. It has also been used in the detection and quantification of miRs in various biological fluids, such as plasma [59] and cerebrospinal fluid [60].

Recently, miRNAs have risen to prominence as biomarkers for many disease states and as tools to assist in medical decisions. Research has indicated the potential use of miR-155 as a biomarker in viral diseases (hepatitis C (HC), chronic hepatitis B (CHB), Japanese encephalitis (JE), and coronavirus disease 2019 (COVID-19)), and to predict their severity, inflammation, and mortality [42,43,44,45,46,47,48,49,50,51]. However, it should be emphasized that these studies were not conducted using dPCR, but quantitative PCR or sequencing, and the research material was not tissue but serum, plasma, and peripheral blood mononuclear cells (PBMCs).

To our knowledge, there are no published studies using dPCR to directly quantify host miRs in liver, lung, kidney, and spleen tissues in viral infection, nor in this particular *L. europaeus* infection. Thus, this method represents a technological advancement in detecting and quantifying miR, especially miR-155 in tissues after viral infection, as well as in virus–host interaction studies. Using the dPCR method, we found that, based on the level of ocu-miR-155-5p expression in organs, disease-changed tissues can be distinguished from the healthy tissues in control rabbits. These results indicate that the expression level of miR-155-5p in these tissues may have a sensitive and specific value as a potential candidate for a tissue biomarker of inflammation in rabbits infected with *L. europaeus* and in diseases of viral etiologies in these organs.

Liver inflammation protects it from infection and injury, but an excessive inflammation may lead to an extensive loss of hepatocytes and, eventually, permanent hepatic damage [83]. The liver microenvironment is rich in many stimuli that can also trigger the expression of miR-155. miR-155 has been shown to be involved in the progression of liver inflammation [8,84]. This molecule is expressed in hepatocytes and endothelial cells, and during inflammation, it is abundantly expressed in inflammatory cells, such as monocytes, NK cells, T lymphocytes and their subpopulations, and B lymphocytes [85]. Moreover, the high expression of miR-155 in the liver tissue can be explained by its macrophage origin. The liver has a large number of Browicz–Kupffer cells. During a local inflammatory reaction, Browicz–Kupffer cells secrete very large quantities of cytokines, e.g., IL-1 and IL-6, which stimulate the production of acute-phase proteins and induce an increase in the expression of miR-155 in the liver tissue [5]. Moreover, hepatic resident T (TCRαβ), Th (CD4), and cytotoxic T (Tc) (CD8) lymphocytes, as well as natural killer (NKT) (CD56) cells and T γδ lymphocytes [5], are also involved in inflammation and are also the origin of miR-155, which, together with neutrophils as inflammatory cells recruited from the blood, infiltrate the liver in *L. europaeus*/RHDV infection [12]. In *L. europaeus*/RHDV infection, the liver is the most damaged organ. It becomes enlarged and inflammatory infiltrates can be observed that are rich in polymorphonuclear (PMN) cells and T and B lymphocytes, as well as the activation of Browicz–Kupffer cells, degenerative changes in hepatocytes consistent with apoptosis and necrosis, and leukopenia [12,19,20,21,36,86,87]. We believe that the increase in ocu-miRNA-155-5p expression in the liver is a marker of inflammation and, at the same time, damage to this organ. The inflammatory response induced a cascade of mediators, such as TNF-α, transforming growth factor β (TGF-β), IL-1β, IL-6, and IFN, as well as TLR ligands (TLR, TLR3, and TLR4) in monocytes and macrophages, which, in turn, induced an increase in miR-155 expression in the liver tissue [39]. O’Toole et al. [37] and Marques et al. [36] showed that, in infection with RHDV2, there is a marked upregulation of the three acute-phase cytokines, IL-6, IL-1β, IL-1, IFN-γ, IL-8, and TNF-α in the liver. The expression of miR-155 is controlled by multiple signaling pathways. Regulatory cytokines including IL-8 and TNF-α can trigger or inhibit miR-155 expression in both liver-resident and infiltrating cells [8,88]. On the other hand, the overexpression of miR-155 can suppresses the production of proinflammatory cytokines [88]. In previous qPCR studies [38], we showed a 27-fold increase in IL-10 expression, which exerted anti-inflammatory properties in the liver tissue of rabbits infected with *L. europaeus*. The increased expression of IL-10 indicates an excessively intense local inflammatory reaction and inhibits the production of cytokines by inflammatory cells infiltrating the tissues [89]. According to one hypothesis, miR-155 may affect the rapid production of high levels of IL-10 as an inhibitor of inflammation [89]. On the other hand, IL-10 can decrease miR-155 expression by inhibiting ETS proto-oncogene 2 and transcription factor (ETS2) [8,88].

The above-mentioned result concerning the expression of ocu-miR-155-5p in the liver tissue determined by dPCR can only be related to the analogous determination of this molecule by RT-qPCR in our earlier studies (a fold-change of 5.8) [39]. Ocu-miR-155-5p in the liver tissue was determined after infection with the RHDV strain Erfurt GI.1a (RHDVa), causing 100% mortality of the infected animals. In contrast, in this study, animals were infected with *L. europaeus* GI.1a/Rossi, which caused 40% mortality. Such differences in the pathogenicity of the strains affect the course and severity of the disease and the type of inflammation (in this study, it was sub-acute inflammation). However, in our previous studies [39], it was acute inflammation. Nonetheless, both studies showed an increase in miR-155-5p expression in the liver tissue, as observed in the deregulation of host miR-155 assayed from different biological material in viral infections [5,42,43,45,46,47,48]. It has been shown that 80% of this deregulation trends upwards and the remaining 20% trends downwards [5]. In addition, the experience with COVID-19 [43,44,48,50,51] indicates that the miR-155 expression pattern can distinguish the acute and sub-acute phases of the disease [43,44,48,51] and predict mortality [50], which is consistent with our previous observations. The expression of miR-155 in the liver tissue in the case of infection with the Erfurt strain (100% mortality) had a fold-change of 5.8 [39], while in the case of the Rossi strain (40% mortality), the fold-change was 2.5.

The expression of miR-155 after infection with *L. europaeus* was the same (a fold-change of 2.1) in the lung and kidney tissues.

MiR-155 is a master regulator of lung diseases, including asthma, cystic fibrosis [8], and COVID-19 [90]. The literature data on the expression of miR-155 in lung tissues in the course of diseases of a viral etiology are very limited [8,90]. There are also no studies on the determination of this molecule’s dPCR methods. It should be emphasized that the highest average copy number (copies/µL) of miR-155-5p in rabbits infected with *L. europaeus* GI.1a/Rossi was recorded in the lungs. The literature indicates that *L. europaeus* infection leads to pathological and histopathological changes in the lungs. Lymphocyte proliferation, granulocyte infiltration, and activation of lung macrophages, as well as hyperemia, pulmonary edema, and interalveolar and perivascular hemorrhages, are noted [12,86,87]. These data indicate inflammation, accompanied by an increase in miR-155 expression in this tissue. It has been shown that the expression of miR-155 in the lungs is rapidly induced following infection since PAMPs can act as inducers of miR-155 expression, and its expression is also induced by TLR agonists. The signals arising from TLR4 and TLR3 can trigger the expression of miR-155 in lung macrophages via Toll/interleukin-1 receptor (TRIF)-dependent and myeloid differentiation primary response 88 (MyD88)-dependent signaling pathways [5]. Pro-inflammatory mediators, such as alarmins, TNF-α, and IL-1β, as well as IFN-β and IFN-γ, can also trigger miR-155 expression in alveolar macrophages. Some transcription factors, in particular, signal transducer and activator of transcription 3 (STAT3), activator protein 1 (AP-1), purine box 1 (PU.1), myeloblastosis (MYB), and Ets2, can all contribute to miR-155 expression [5]. These resident tissue cells, as well as inflammatory cells (granulocytes and monocytes), recruited from the blood and infiltrating the lungs in the course of RHDV, may be the source of miR-155 in response to a wide range of inflammatory mediators and activation of inflammatory pathways, including NF-ĸB [83].

Our results indicate that the expression level of miR-155-5p in the lungs may be sensitive and specific enough to have value as a potential candidate for a biomarker of inflammation in *L. europaeus* infection. Gaytan-Pacheco et al. [90] demonstrated that miR-155, miR-146a, and miR-221 are involved in the inflammatory immune response in severe COVID-19 patients. The plasmatic expression of circulating miRNAs involved in inflammatory and pathological host immune responses was assessed using RT-qPCR. Compared with the controls, a significant upregulation of miR-155 was observed.

Kidney inflammation contributes to progressive renal injury, which may lead to glomerulonephritis, end-stage renal disease, or acute or chronic kidney disease (CKD). Stimuli that can induce kidney injury activate transcription factors NF-κB, which induce expression of miR-155 in the kidney tissue environment [83]. In *L. europaeus*/RHDV infection, the kidneys are the organ with granulocytic and lymphocytic infiltrates, enlargement with dark red spots, hyperemia, hemorrhages, dilated renal tubules, and degeneration of the renal tubular epithelium [12,87]. The role of miR-155 in viral nephritis is unknown. It can indicate that the source of miR-155 in this tissue may be resident, and inflammatory cells (granulocytes, monocytes, and lymphocytes) recruited from the blood infiltrate into the kidneys during RHD [83]. Wang et al. [91] proposed circulating miR-155 and miR-126 as biomarkers of inflammation in patients with end-stage renal disease (ESRD). The results of these studies are different from ours. In [91], miR-155 and miR-126 were assessed in the plasma of 30 ESRD patients and 20 healthy controls using qRT-PCR. The circulating levels of miR-155 and miR-126 were significantly reduced in patients with ESRD compared to the healthy controls. The results indicate that circulating miR-155 and miR-126 might be involved in the development of ESRD, and a reduced amount of circulating miR-126 and miR-155 may be associated with an increased rate of miRNA degradation in patients with ESRD.

A decrease in ocu-miR-155-5p expression in infected rabbits was demonstrated in the spleen (a fold-change of 0.6, *p*-value = 0.002).

The spleen is the main lymphatic organ and is responsible for the host’s systemic response. It is extremely important in defending the body against viral infections [92,93]. In RHDV infection, the enlargement of the spleen (splenomegaly), activated macrophages (macrophage hyperplasia) displaying active phagocytosis, mottled dark red discoloration, T lymphocytes, hyperemia, and leukopenia are observed. In the white pulp, there is a moderate to severe decrease in the proportion of lymphocytes to macrophages, reflecting general lymphoid depletion in comparison with the control animals [12,36,86,87]. The healthy spleen tissue is a cell-rich environment and miR-155 expression in this environment is rapidly induced by PAMP [94]. The research by O’Toole et al. [37] and Marques et al. [36] showed that, in an infection with RHDV2, there is a marked upregulation of the three acute-phase cytokines IL-6, IL-1β, IL-1, IFN-γ, IL-8, and TNF-α in the spleen [37]. These cytokines can trigger the expression of miR-155 in both spleen-resident and infiltrating cells. In the course of infection with *L. europaeus*, inflammatory and degenerative changes in the spleen are also observed, which is probably associated with a decrease/depletion in B and T lymphocytes, as well as T and B lymphocytes and their subpopulations in the peripheral blood, which results in a weaker response from the immune system and thus a decrease in miR-155 expression in the pathological spleen [12,14,17,18]. Teixeira et al. [27] showed that the number of CD4+Foxp3+ Tregs was reduced in the spleen of adult rabbits 24 h after infection with RHDV. Because Tregs reduce inflammation, the authors believe that their decline may contribute to the natural susceptibility of adult rabbits to RHDV infection. On the other hand, increasing evidence has suggested that the number of Treg cells in the spleen is reduced in miR-155-deficient mice [95]. This finding requires further research. It should also be emphasized that, in previous studies using the qPCR method [38], we showed a 38-fold increase in the expression of anti-inflammatory IL-10 in the spleen tissue of rabbits infected with *L. europaeus*, which proves an excessively intense local inflammatory reaction in this organ. Similarly, Trzeciak-Ryczek et al. [33] showed an increased expression of IL-10 in peripheral blood leukocytes (PBLs) in rabbits infected with RHDV. According to one hypothesis [5], the overexpression of IL-10 inhibits TLR4-dependent signaling and reduces miR-155 expression in a STAT3-dependent manner in the spleen microenvironment. The decreased expression of miR-155 in the spleen has also been reported in the case of infection with lymphotropic viruses [4,96,97]. In deep-sequencing studies of Marek’s disease virus (MDV)-induced splenic tumors, Burnside et al. [96] reported decreased levels of miR-155, compared to the normal spleen, resting T cells, or activated T cells. These observations were confirmed by the in vitro study of Yao et al. [97], who observed a down-regulation of miR-155 specific to MDV-transformed tumor cells. In addition, the level of miR-155 expression was consistently reduced in all tested MDV-1-transformed lymphoblastoid cell lines, demonstrating that the down-regulation of miR-155 is a feature unique to the MDV transformation of T cells.

## 5. Conclusions

In this study, we introduced a digital PCR method using the QIAcuity Digital PCR System (Qiagen, Hilden, Germany) for the sensitive quantification of miR-155-5p in host tissues with *L. europaeus* infection for the first time. In the context of viral infections, this is the first report on technological progress in the detection and quantification of miR in tissues, as well as in virus–host interaction studies.

We found a significantly altered expression of miR-155 in infected rabbit tissues compared to the healthy control rabbits, making it a potential candidate for a tissue biomarker of inflammation in the infection of *L. europaeus*. In this paper, we indicated that *L. europaeus* infection in rabbits is a good research model and a signpost for the search for miRNAs as potential candidates for tissue biomarkers of inflammation in viral infections. Perhaps, in the future, in the infectious diseases of humans and animals, it will be possible to recognize and monitor inflammation and the effectiveness of treatment, based on the absolute level of miRs in tissues, serum, and plasma using the dPCR method. One of the limitations of the method is its invasiveness, as well as the need for performing a biopsy in animals and humans. We presented arguments for this circumstance in the introduction. We believe that the benefits of using miR-155 as a biomarker outweigh the potential risk of complications after biopsy. A biopsy in the diagnostic procedure, if needed, is not a problem since the tissue material is taken regardless. In addition, the expression of miR-155 in specific organs provides the opportunity to examine the local and current inflammatory response in them, rather than using the less-specific circulating miR-155, which can be a more significant advantage of this tissue’s potential biomarkers.

More research is needed on miR-155-5p and other inflammation-related miRNAs to confirm their function as a biomarker in animals and humans. Our report presents a new path in discovering potential candidates for tissue biomarkers of inflammation.

## Data Availability

Data are available after personal email request from Beata Hukowska-Szematowicz (beata.hukowska-szematowicz@usz.edu.pl).
